# Factors Predicting Reversion from Mild Cognitive Impairment to Normal Cognitive Functioning: A Population-Based Study

**DOI:** 10.1371/journal.pone.0059649

**Published:** 2013-03-27

**Authors:** Perminder S. Sachdev, Darren M. Lipnicki, John Crawford, Simone Reppermund, Nicole A. Kochan, Julian N. Trollor, Wei Wen, Brian Draper, Melissa J. Slavin, Kristan Kang, Ora Lux, Karen A. Mather, Henry Brodaty, the Sydney Memory, Ageing Study Team

**Affiliations:** 1 Centre for Healthy Brain Ageing, School of Psychiatry, University of New South Wales Medicine, Sydney, New South Wales, Australia; 2 Neuropsychiatric Institute, Prince of Wales Hospital, Sydney, New South Wales, Australia; 3 Primary Dementia Collaborative Research Centre, School of Psychiatry, University of New South Wales Medicine, Sydney, New South Wales, Australia; 4 Academic Department for Old Age Psychiatry, Prince of Wales Hospital, Sydney, New South Wales, Australia; 5 Department of Developmental Disability Neuropsychiatry, School of Psychiatry, University of New South Wales Medicine, Sydney, New South Wales, Australia; 6 South-Eastern Area Laboratory Services, Prince of Wales Hospital, Sydney, New South Wales, Australia; Nathan Kline Institute and New York University School of Medicine, United States of America

## Abstract

**Introduction:**

Mild cognitive impairment (MCI) is associated with an increased risk of developing dementia. However, many individuals diagnosed with MCI are found to have reverted to normal cognition on follow-up. This study investigated factors predicting or associated with reversion from MCI to normal cognition.

**Methods:**

Our analyses considered 223 participants (48.9% male) aged 71–89 years, drawn from the prospective, population-based Sydney Memory and Ageing Study. All were diagnosed with MCI at baseline and subsequently classified with either normal cognition or repeat diagnosis of MCI after two years (a further 11 participants who progressed from MCI to dementia were excluded). Associations with reversion were investigated for (1) baseline factors that included diagnostic features, personality, neuroimaging, sociodemographics, lifestyle, and physical and mental health; (2) longitudinal change in potentially modifiable factors.

**Results:**

There were 66 reverters to normal cognition and 157 non-reverters (stable MCI). Regression analyses identified diagnostic features as most predictive of prognosis, with reversion less likely in participants with multiple-domain MCI (*p* = 0.011), a moderately or severely impaired cognitive domain (*p* = 0.002 and *p* = 0.006), or an informant-based memory complaint (*p* = 0.031). Reversion was also less likely for participants with arthritis (*p* = 0.037), but more likely for participants with higher complex mental activity (*p* = 0.003), greater openness to experience (*p* = 0.041), better vision (*p* = 0.014), better smelling ability (*p* = 0.040), or larger combined volume of the left hippocampus and left amygdala (*p*<0.040). Reversion was also associated with a larger drop in diastolic blood pressure between baseline and follow-up (*p* = 0.026).

**Discussion:**

Numerous factors are associated with reversion from MCI to normal cognition. Assessing these factors could facilitate more accurate prognosis of individuals with MCI. Participation in cognitively enriching activities and efforts to lower blood pressure might promote reversion.

## Introduction

The diagnosis of mild cognitive impairment (MCI) is increasingly being used in epidemiological studies of cognitive disorders as well as in the clinic [1]. As a nosological entity, MCI conveys important health implications, in particular an increased risk of developing dementia in the near future. This is evident from studies of MCI patients presenting to memory disorders clinics, in whom the annual rate of progression to dementia is reported to be between 10% and 15% [2]. While rates of progression are lower in population-based studies, between 6% and 10%, these are still higher than the 1% to 2% annualised incidence rates of dementia and Alzheimer’s disease (AD) in the general older population [1].

The significance of an MCI categorisation sits uneasily with longitudinal data suggesting that MCI may be unstable, with many individuals found to be cognitively normal on follow-up [3]–[6]. The rates of reversion to normal vary from 4.5% [7] to as high as 53% [3]. A number of explanations can account for this variability, including individuals with normal cognition on follow-up having been initially misdiagnosed with MCI due to the use of very liberal criteria [3], [8] or inappropriate normative neuropsychological data against which the individual’s performance was compared [8]. It is also possible that MCI was diagnosed during a temporary decline in cognitive functioning associated with depression [9], mild psychiatric conditions or stress [10], or on the basis of poor cognitive test performance arising from general ill-health or poor motivation [11]. It has also been suggested that ‘unstable’ MCI represents a pre-MCI condition that will develop into ‘stable’ MCI with time, prior to which cognitive impairment is subtle and only manifests under certain circumstances [12]. There are some causes of MCI that are truly reversible, such as metabolic disorders and deficiency syndromes, and others with an acute phase of impairment that subsequently improves, including traumatic brain injury, substance use and cerebrovascular events. Finally, it is possible that some individuals with MCI improve because of pharmacological intervention or lifestyle change, including increased cognitive and physical activity and reduced stress [10].

The health and social implications of an MCI diagnosis make it important to identify factors indicative of a good prognosis. While a number of factors used to diagnose MCI are reportedly associated with reversion [3], [10], [13]–[18], non-diagnostic factors have received relatively little attention [10], [14], [15]. There are numerous factors associated with cognition in the elderly not used in diagnosing MCI, and many of these could help in predicting reversion to normal cognition and/or be suitable targets for remedial strategies. The aim of the present study was to identify factors associated with reversion from MCI to normal cognition from among a broad range of factor types, including sociodemographic, neuroimaging, lifestyle, physical and mental health, diagnostic, and personality characteristics.

## Methods

### Participants

Participants were from the Sydney Memory and Ageing Study (MAS), a longitudinal study of community-dwelling individuals aged 70 to 90 years recruited randomly from areas of Sydney, Australia, through the electoral roll. A full description of the recruitment procedures has been previously published [19]. Participants were excluded if they had a previous diagnosis of dementia, psychotic symptoms or a diagnosis of schizophrenia or bipolar disorder, multiple sclerosis, motor neuron disease, developmental disability, progressive malignancy, or if they had medical or psychological conditions that may have prevented them from completing assessments. Participants were also excluded if they had a Mini-Mental State Examination (MMSE) [20] score of <24 adjusted for age, education and non-English speaking background [21] at study entry, or if they had received a diagnosis of dementia after comprehensive baseline assessment. The representativeness of the MAS sample was assessed through comparisons with geographically-relevant census data [22].

**Figure 1:**
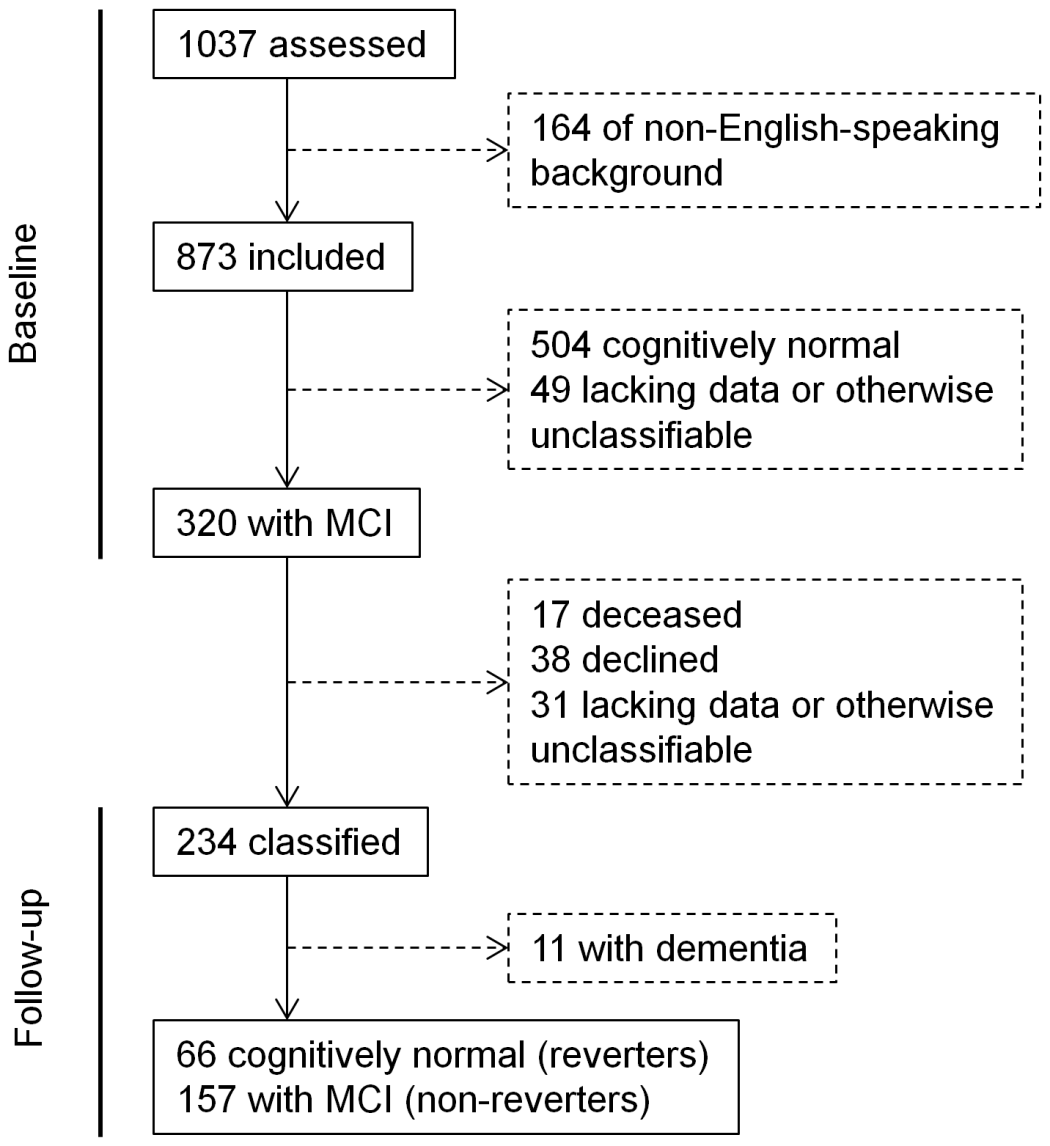
Flow diagram of sample selection.

Figure 1 depicts the recruitment and selection process for the study. The total MAS sample comprised 1037 participants. We excluded 164 individuals deemed to be not of English-speaking background (English acquired after 10 years of age) because neuropsychological test norms for this group are lacking. There were 320 participants from the remaining 873 diagnosed with MCI at baseline.

### Ethics Statement

The study was approved by the ethics committees of the University of New South Wales and the South Eastern Sydney and Illawarra Area Health Service, and each participant gave written informed consent.

### MCI Diagnoses

Participants underwent face-to-face neuropsychological assessments by trained psychology graduates, using a battery of tests to assess functioning within five cognitive domains: memory, language, attention/processing speed, visuospatial and executive functioning (see Table S1). Consensus diagnoses of MCI were made by a panel of psychogeriatricians, neuropsychiatrists and clinical and research neuropsychologists using current international consensus criteria [23]. MCI was diagnosed in individuals who met all of the following: self or informant complaint of decline in memory or other cognitive function; cognitive impairment on testing (performance on at least one test measure 1.5 SD or more below published normative values, with adjustment for age and/or education where possible); no dementia on the basis of DSM-IV criteria [24]; and no or minimal impairment in instrumental activities of daily living attributable to cognitive impairment (total average score <3.0 on the Bayer Activity of Daily Living (ADL) Scale [25] adjusted for physical impairment). The test administrators and clinical consensus panel were blind to baseline diagnoses when making assessments and diagnoses at follow-up.

### Measures and Criteria for Categorical Classifications

Measures were obtained in part from interviews and questionnaires addressing sociodemographic factors, lifestyle, and aspects of cardiac, physical, mental and general health (listed in Table 1). This included the Goldberg Anxiety Scale [26], and most participants (93.9%) had an informant who completed a phone interview and additional questionnaires, including the Bayer ADL Scale. The 15-item version of the Geriatric Depression Scale [27] and the neuroticism, openness, and conscientiousness scales of the NEO-Five Factor Inventory [28] were given to participants for self-completion and return by mail. The personality items were added to the Sydney MAS after the study began, and thus not received by all participants.

**Table 1:** Sociodemographic, lifestyle and health characteristics of reverters and non-reverters at baseline^a^.

Factor	Reverters	Non-reverters	*p* value
	(n = 66)^b^	(n = 157)^b^	
***Sociodemographic***			
Age, mean (SD), y	78.61 (4.47)	78.48 (4.45)	.847
Males	34 (51.5)	75 (47.8)	.610
Education, mean (SD), y	12.29 (3.94)	11.23 (3.53)	.049
Married or de facto	24 (36.9)	71 (45.2)	.255
***Cardiac Health***			
Hypertension	52 (78.8)	130 (82.8)	.480
Antihypertensives	37 (56.1)	97 (61.8)	.426
Coronary artery disease	14 (21.2)	27 (17.2)	.480
Atrial fibrillation	5 (7.8)	11 (7.1)	.863
Other heart disease^c^	7 (10.6)	21 (13.4)	.569
Systolic BP, mean (SD), mmHg	145.72 (19.00)	144.05 (20.16)	.573
Diastolic BP, mean (SD), mmHg	82.83 (10.81)	81.46 (9.39)	.353
***Physical Health***			
BMI, mean (SD), kg/m^2^	26.57 (3.99)	27.24 (4.56)	.309
Diabetes	10 (15.2)	20 (12.7)	.630
Hypoglycemics	5 (7.6)	14 (8.9)	.743
High cholesterol diagnosis	37 (56.1)	88 (56.1)	.999
Hypolipidemics	33 (50.0)	71 (45.2)	.514
Stroke	4 (6.1)	5 (3.2)	.324
Migraines	6 (9.1)	22 (14.0)	.311
Kidney disease	1 (1.5)	7 (4.5)	.277
Arthritis	29 (45.3)	97 (61.8)	.025
Apnea	4 (6.1)	6 (3.8)	.467
Anemia	7 (10.6)	19 (12.3)	.727
***Mental Health***			
GDS score, mean (SD)	2.30 (1.94)	2.18 (1.81)	.659
History of depression	9 (13.6)	24 (15.3)	.751
GAS score, mean (SD)	1.0 (1.8)	1.4 (2.2)	.170
Antidepressants	5 (7.6)	15 (9.6)	.637
Antianxiety agents	1 (1.5)	10 (6.4)	.127
***Lifestyle***			
Alcohol consumption			.486
Abstainer	7 (10.6)	13 (8.3)	
≤1 drink/day	36 (54.5)	76 (48.4)	
>1 drink/day	23 (34.8)	68 (43.3)	
Smoking			.974
Never	32 (48.5)	74 (47.1)	
Past	31 (47.0)	75 (47.8)	
Current	3 (4.5)	8 (5.1)	
Mental activity, mean (SD)^d^	2.70 (0.82)	2.26 (0.84)	<.001
Physical activity, mean (SD)^e^	1.52 (0.96)	1.65 (1.11)	.385
Social activity			.249
<5 (contacts/month)	6 (9.4)	22 (14.2)	
5–10 (contacts/month)	12 (18.8)	40 (25.8)	
>10 (contacts/month)	46 (71.9)	93 (60.0)	
***General Health***			
Self-reported			.268
Poor to fair	7 (10.6)	21 (13.4)	
Good	23 (34.8)	69 (43.9)	
Very good to excellent	36 (54.5)	67 (42.7)	
6-m walk time, mean (SD), s	9.10 (2.73)	9.58 (2.86)	.255
BSIT score, mean (SD)	9.52 (2.09)	8.90 (2.19)	.054
Visual acuity, mean (SD)^f^	0.71 (0.19)	0.63 (0.20)	.014
***Laboratory Measures***			
Apolipoprotein E ε4 allele	12 (19.4)	49 (32.7)	.051
Homocysteine, mean (SD), umol/L	11.46 (5.02)	12.46 (4.51)	.164
Cholesterol, mean (SD), mmol/L	4.59 (0.96)	4.82 (1.09)	.150
eGFR <60 ml/min/1.73 m^2^	20 (32.8)	66 (43.1)	.163

BMI = body mass index; BP = blood pressure; BSIT = Brief Smell Identification Test; eGFR = estimated glomerular filtration rate; GAS = Goldberg Anxiety Scale; GDS = Geriatric Depression Scale.

a Data presented as No. (%) unless otherwise indicated.

b Maximum n, with small amounts of missing data for some factors.

c Any of cardiac arrhythmia, cardiomyopathy, or heart valve disease.

d Average days/week of participation in mental activities.

e Number of different physical activities participated in.

f Arbitrary units, averaged for the two eyes.

A brief physical examination conducted by a trained research assistant included measures of seated blood pressure (BP), height and weight, a 6-metre timed walk [29], the Brief Smell Identification Test (BSIT) [30], and a corrected vision test using a 3-metre Standard Contrast LogMAR chart. Venous blood was collected following an overnight fast, and lithium heparin, EDTA plasma and serum aliquots frozen at –80°C. Total cholesterol was measured in heparin plasma aliquots using a Beckman LX20 Analyser by a timed-endpoint method (Fullerton, California, USA), and homocysteine levels determined from EDTA plasma aliquots using reverse phase HPLC with fluorometric detection after derivatization with 4-aminosulfonyl-7-fluorobenzo-2-oxa1,3-diazole (CV 6.7% at 11.7 umol/L, 6% at 30.0 umol/L) (BioRad Munich, Germany). *APOE* genotyping was performed using standard procedures, as described previously [19].

Around half (52.3%) of all MAS participants consented to an MRI scan, which was performed on either a Philips 3 T *Achieva* Quasar Dual scanner or a Philips 3 T *Integra* Quasar Dual scanner (Philips Medical System, Best, The Netherlands). With both scanners, T1-weighted structural images were acquired using the turbo field echo sequence: TR = 6.39 ms, TE = 2.9 ms, flip angle = 8°, matrix size = 256 × 256, FOV = 256 × 256 × 190, and slice thickness = 1 mm with no gap between; yielding 1 × 1 × 1 mm^3^ isotropic voxels. T2-weighted fluid attenuated inversion recovery images were also acquired, to evaluate white matter hyperintensities. The sequence parameters were: TR = 10000 ms, TE = 110 ms, TI = 2800; matrix size = 512 × 512; slice thickness = 3.5 mm with no gap between slices; yielding a spatial resolution of 0.488 × 0.488 × 3.5 mm^3^/voxel. Statistical Parametric Mapping 5 (SPM5) software (Wellcome Trust Centre for Neuroimaging, UK) was used to process and analyse the T1 images. Briefly, the major steps included: (1) segmentation of T1 images into grey matter, white matter, and cerebrospinal fluid; (2) registration of grey and white matter maps to our group average template, using the DARTEL toolbox [31] in SPM5 to obtain the DARTEL flow fields; (3) each individual’s DARTEL flow field was applied to the automated anatomical labeling template [32] in Montreal Neurological Institute space to create each subject’s grey matter region of interest mask; (4) grey matter volumes of each region of interest (see Table S2) were calculated from the warped automated anatomical labeling mask. Total brain volume was calculated as the sum of grey and white matter, and intracranial volume as the sum of total brain volume and cerebrospinal fluid. The total volume of white matter hyperintensities for each participant was determined using a computer algorithm described in detail elsewhere [33].

Participants were classified as hypertensive if meeting one of: previous diagnosis and current treatment, or either systolic BP≥140 mmHg or diastolic BP≥90 mmHg (as per JNC-7 values) [34]. A self-reported previous diagnosis of heart attack or angina was taken as coronary artery disease, and of cardiac arrhythmia, cardiomyopathy or heart valve disease as ‘other heart disease’. History of depression reflected self-reports of both a previous diagnosis and treatment. Current mental activity was calculated as the average days/week of participation in 13 activities (e.g., reading books); physical activity as the sum of participation across eight listed activities (e.g., bicycling), a valid other reported activity (e.g., yoga), and walking; and social activity as the average number of face-to-face contacts with friends or relatives per month. Alcohol consumption was measured in terms of the average number of standard drinks (10 g alcohol) per day over the past year. Visual acuity was calculated as 1.78 minus log_10_(line number) and averaged across eyes.

MCI was subtyped as either amnestic or non-amnestic (depending on whether or not objective testing revealed a memory impairment), and as either single- or multiple-domain (given the number of cognitive domains with impairment) [23]. We also calculated Z scores reflecting a participant’s overall performance in each cognitive domain relative to the study sample, adjusted for age, education, and sex [8]. Across all domains, the participant’s worst level of performance was categorised as low (above −1.0 SD), mildly impaired (between −1.0 inclusive and −1.5 SD), moderately impaired (between −1.5 inclusive and −2.0 SD), or severely impaired (equal to or less than −2.0 SD).

### Missing Participants

Figure 1 shows that 86 of the 320 individuals with MCI at baseline were missing a classification at follow-up: 17 were deceased, 38 declined, and 31 could not be reliably diagnosed (primarily due to insufficient neuropsychological data). Compared to the 234 individuals with a follow-up classification, those without were significantly older, had lower systolic BP, consumed less alcohol, and reported less participation in mental and physical activities and lower levels of self-rated health. The missing individuals also had lower MMSE scores, a higher proportion of informant-based non-memory complaints, and higher neuroticism scores (see Table S3).

### Statistical Analysis

Descriptive statistics were computed for the participants who reverted to normal cognition upon follow-up and those who did not (i.e., those with stable MCI or who had progressed to dementia). For all measures, we first conducted simple comparisons between reverters and non-reverters using either *t*- or *χ* ^2^ tests. We then performed logistic regressions to identify measures that individually discriminated between reverters and non-reverters at a significance level of *p<*0.10 when controlling for age and sex (and intracranial volume for neuroimaging data). Each discriminating measure was assigned to one of six sets: cognitive reserve, sensory, health and genetic, neuroimaging, personality, and diagnostic. For each of these sets we then performed a separate multivariable logistic regression featuring the discriminating measures assigned to that particular set. For example, the regression for the cognitive reserve set featured education and mental activity, whereas that for the sensory set featured BSIT score and visual acuity. All regressions were controlled for age and sex (and intracranial volume for the neuroimaging set). We did not attempt a comprehensive multivariable regression containing the discriminating variables from all six sets. This was because only subgroups of our sample had neuroimaging or personality scale data, limiting the number of participants with data for all discriminating measures and leaving us with an events per variable value that prevented a valid overall regression from being performed (as per [35]).

The analyses of baseline predictors were supplemented by an investigation of associations between reversion and longitudinal change over the follow-up interval in factors potentially modifiable by lifestyle alteration, medication, or other intervention. Blood pressure, body mass index, depression, cholesterol and homocysteine levels, alcohol consumption, and mental, physical, and social activity were analysed, but smoking was not included as there were very few changes in status (3.4% of participants). Descriptive statistics were computed and reverters and non-reverters compared, first with ANOVA or *χ* ^2^ tests and then with logistic regressions controlling for age and sex.

All analyses were performed using IBM SPSS Statistics 20, and our final conclusions were based on regression outcomes where *p*<0.05.

## Results

### Final Sample of Reverters and Non-reverters

For the 234 individuals with classifications at both baseline and follow-up, the duration between these time-points ranged from 17.1 to 29.9 months (mean ± SD = 23.0±1.5). As shown in Figure 1, 66 were classified at follow-up as having normal cognition, 157 were re-diagnosed with MCI, and 11 had developed dementia. We excluded the individuals with dementia from any further analyses, meaning our non-reverter group was comprised only of individuals with stable MCI. The final number of participants included in our sample of reverters and non-reverters was 223; their ages ranged from 71 to 89 years (mean ± SD = 78.52±4.45), and 48.9% were male.

### Simple Comparisons of Reverters and Non-reverters

As shown in Table 1, there were only a few sociodemographic, health and lifestyle characteristics on which reverters and non-reverters differed significantly (*p*<0.05). There was no difference in age, but reverters were more mentally active and had more years of education. The two groups did not differ in terms of cardiovascular risk factors or indicators of physical health, except for lower rates of arthritis and better visual acuity in reverters. Statistical trends (*p*<0.10) also favoured reverters as having better smelling ability (BSIT scores) and a decreased likelihood of an *APOE* ε4 allele. Mental health status, in terms of depression, anxiety and use of psychotropic drugs, were not discrepant.

Table 2 shows baseline diagnostic and neuroimaging characteristics and personality scale scores of reverters and non-reverters. Reverters had higher MMSE scores, whereas rates of moderate or severe impairment of a cognitive domain were higher in non-reverters. The pattern of informant versus self-reported memory complaints and rates of multiple-domain MCI also differed between reverters and non-reverters, but the pattern of non-memory complaints and rates of amnestic MCI did not. Personality scale score differences included lower levels of neuroticism and greater levels of openness in reverters than non-reverters. There was no difference in conscientiousness between these groups. Reverters had greater white matter volumes, which combined with a trend towards greater grey matter volumes led to them also having significantly greater total brain volumes. In contrast, intracranial volume, a measure of premorbid brain size, did not differ between reverters and non-reverters. There were many regions of interest for which volume was greater in reverters than non-reverters, most notably the hippocampus and amygdala of the left hemisphere (Table 2 only shows regions of interest that were significantly different with adjusted comparisons; see Table S2 for a full list).

**Table 2:** Baseline diagnostic characteristics, brain region volumes and personality scale scores of reverters and non-reverters^a^.

Factor	Reverters	Non-reverters	*p* value
***Diagnostic characteristics***	**(n = 66)** b	**(n = 157)** b	
MMSE score^c^	28.8 (1.3)	28.0 (1.5)	.001
Bayer ADL Scale score	1.5 (0.6)	1.6 (0.6)	.268
Memory complaint			.038
Informant, No. (%)	39 (61.9)	108 (76.1)	
Self-report only, No. (%)	24 (38.1)	34 (23.9)	
Non-memory complaint			.886
Informant, No. (%)	20 (40.8)	50 (42.0)	
Self-report only, No. (%)	29 (59.2)	69 (58.0)	
Amnestic MCI			.252
No, No. (%)	27 (41.5)	77 (50.0)	
Yes, No. (%)	38 (58.5)	77 (50.0)	
Multiple-domain MCI			<.001
No, No. (%)	58 (89.2)	90 (59.2)	
Yes, No. (%)	7 (10.8)	62 (40.8)	
Performance in worst domain			<.001
Low, No. (%)	30 (45.5)	24 (16.7)	
Mildly impaired, No. (%)	25 (37.9)	42 (29.2)	
Moderately impaired, No. (%)	9 (13.6)	48 (33.3)	
Severely impaired, No. (%)	2 (3.0)	30 (20.8)	
***Brain region volumes***	**(n = 37)**	**(n = 92)**	
Grey matter, l	0.756 (0.080)	0.726 (0.109)	.086^d^
White matter, l	0.387 (0.038)	0.371 (0.042)	.045
Total brain volume, l	1.144 (0.101)	1.097 (0.137)	.037^d^
Cerebrospinal fluid, l	0.441 (0.129)	0.444 (0.122)	.871
Intracranial volume, l	1.584 (0.184)	1.542 (0.201)	.269
WMH, mm^3^	13977 (26864)	9330 (11776)	.317^d^
Region of interest,^e^ mm^3^			
Hippocampus (left)	3519 (365)	3281 (451)	.005
Amygdala (left)	868 (112)	787 (125)	.001
Caudate (left)	3278 (455)	3006 (572)	.011
Caudate (right)	3399 (462)	3153 (550)	.018
Putamen (left)	2662 (579)	2423 (505)	.021
Cerebellum 7b (right)	1814 (279)	1683 (282)	.017
Cerebellum 8 (right)	6528 (1421)	6099 (948)	.021
***Personality scale scores***	**(n = 39)**	**(n = 96)**	
Neuroticism	12.1 (8.2)	15.2 (5.7)	.034^d^
Openness	28.3 (6.3)	25.4 (5.5)	.009
Conscientiousness	34.4 (6.1)	33.6 (5.7)	.453

ADL = Activity of Daily Living; MCI = mild cognitive impairment; MMSE = Mini-Mental State Examination; WMH = white matter hyperintensities.

a Data presented as mean (SD) unless stated otherwise.

b Maximum n, with small amounts of missing data for some factors.

c Adjusted for age and education.

d Result for *t*-test for unequal variances.

e Full list of regions of interest in Table S2.

### Adjusted Comparisons of Reverters and Non-reverters

For the sociodemographic, health and lifestyle, diagnostic, and personality variables, the results of univariate logistic regression analyses controlling for age and sex (Table 3) were mostly very similar to the results found using simple comparisons. The only discrepancies in the adjusted analyses were trends for more education (initially significantly) and lower homocysteine levels in reverters. However, for the neuroimaging data, many of the variables found to differ significantly between reverters and non-reverters with simple comparisons no longer discriminated between these groups when age, sex and intracranial volume were controlled for. This outcome is most likely contributed to by the greater proportion of males in the reverter group (a difference evident as a statistical trend).

**Table 3:** Baseline factors associated with reversion from MCI to normal cognition.

Factor	Univariate		Multivariable^a^	
	OR (95% CI)	*p* value	OR (95% CI)	*p* value
***Cognitive reserve***				
Education	1.08 (1.00–1.17)	.061	1.05 (0.96–1.14)	.292
Mental activity	1.90 (1.31–2.74)	.001	1.79 (1.22–2.62)	.003
***Sensory***				
BSIT score	1.19 (1.01–1.39)	.034	1.19 (1.01–1.40)	.040
Visual acuity	9.17 (1.56–53.94)	.014	9.35 (1.56–55.86)	.014
***Health and Genetic***				
Arthritis	0.51 (0.28–0.92)	.025	0.51 (0.27–0.96)	.037
Homocysteine	0.93 (0.86–1.01)	.097	0.93 (0.86–1.01)	.096
Apolipoprotein E ε4 allele	0.48 (0.24–1.00)	.049	0.48 (0.22–1.03)	.058
***Neuroimaging***				
Hippocampus (left)	1.001 (1.000–1.002)	.040	0.999 (0.997–1.002)	.622
Amygdala (left)	1.005 (1.001–1.009)	.011	1.005 (0.998–1.013)	.162
Caudate (left)	1.001 (1.000–1.002)	.052	1.000 (0.998–1.002)	.928
Caudate (right)	1.001 (1.000–1.002)	.074	1.000 (0.998–1.002)	.850
Putamen (left)	1.001 (1.000–1.001)	.088	1.000 (0.999–1.001)	.754
Cerebellum 7b (right)	1.001 (1.000–1.003)	.081	1.001 (0.998–1.004)	.601
Cerebellum 8 (right)	1.000 (1.000–1.001)	.097	1.000 (0.999–1.001)	.858
***Personality***				
Neuroticism scale score	0.93 (0.87–0.99)	.022	0.94 (0.89–1.01)	.078
Openness scale score	1.09 (1.02–1.17)	.012	1.08 (1.00–1.15)	.041
***Diagnostic***				
Informant memory complaint	0.50 (0.26–0.95)	.033	0.44 (0.21–0.93)	.031
Performance in worst domain				
Low	Reference	–	Reference	–
Mildly impaired	0.49 (0.24–1.02)	.057	0.58 (0.26–1.29)	.182
Moderately impaired	0.15 (0.06–0.36)	<.001	0.20 (0.08–0.55)	.002
Severely impaired	0.05 (0.01–0.24)	<.001	0.10 (0.02–0.52)	.006
MMSE score	1.48 (1.17–1.87)	.001	1.23 (0.96–1.59)	.102
Multiple-domain MCI	0.17 (0.07–0.40)	<.001	0.27 (0.10–0.75)	.011

BSIT = Brief Smell Identification Test; CI = confidence interval; MCI = mild cognitive impairment; MMSE = Mini-Mental State Examination; OR = odds ratio.

a Six multivariable regressions were conducted, one for each of the sets of variables labelled cognitive reserve, sensory, health and genetic, neuroimaging, personality, and diagnostic. For example, the regression for the cognitive reserve set featured education and mental activity, whereas that for the sensory set featured BSIT score and visual acuity. All ORs are adjusted for age and sex (neuroimaging results are also adjusted for intracranial volume).

**Figure 2:**
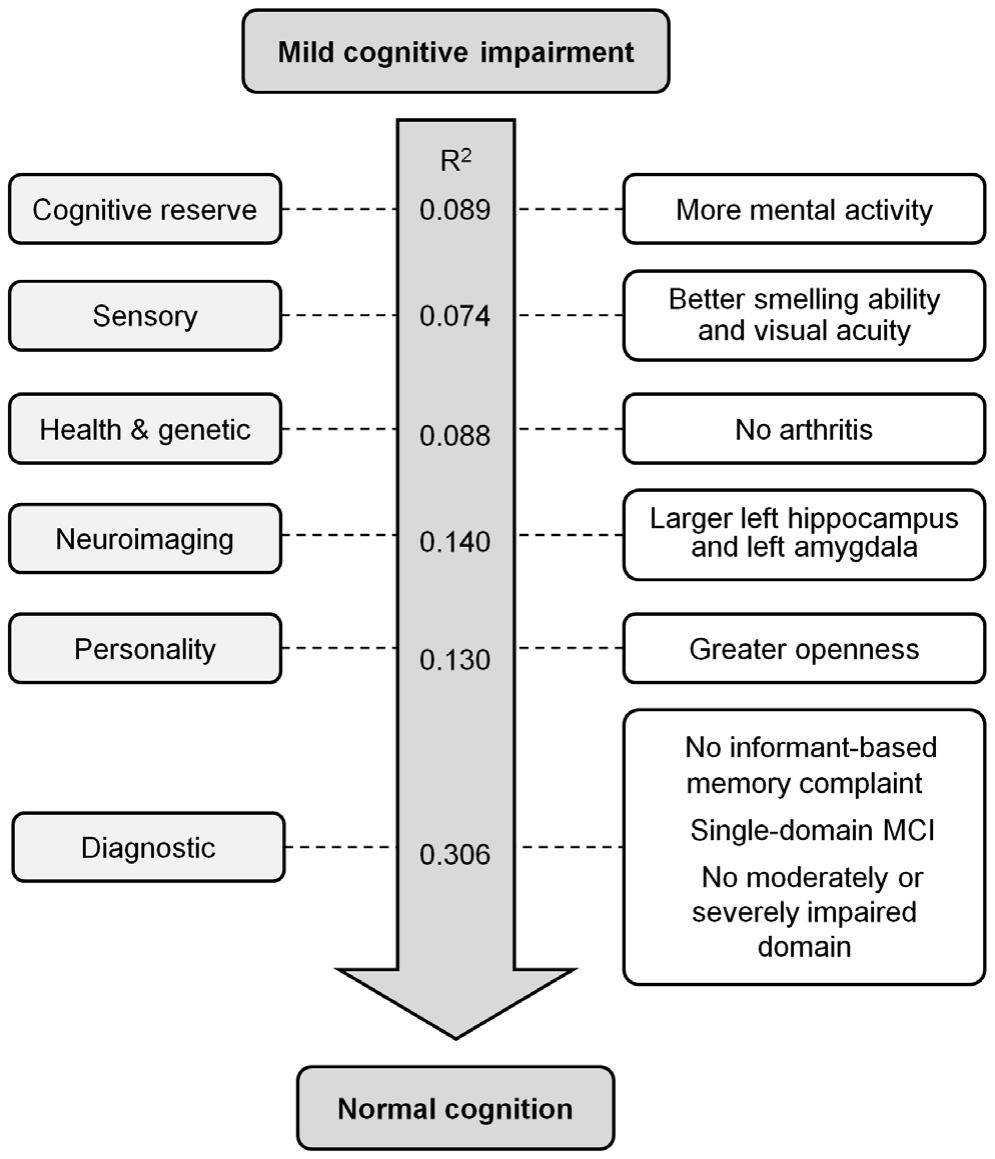
Baseline factors associated with reversion from mild cognitive impairment to normal cognitive functioning. Univariate analyses identified measures that discriminated between reverters and non-reverters. Each of these measures was assigned to one of six sets of related variables: cognitive reserve, sensory, health and genetic, neuroimaging, personality, and diagnostic. For each of these sets we performed a separate multivariable regression containing the discriminating measures assigned to that set, controlling for age and sex (and intracranial volume for the neuroimaging set). A separate Nagelkerke R2 value is shown for each of the six sets. The factors on the right hand side are those from among the variables in the relevant set that were independently associated with reversion (p<0.05).

Table 3 also shows the results of six multivariable logistic regressions, one for each of the six sets of discriminating measures: cognitive reserve, sensory, health and genetic, neuroimaging, personality, and diagnostic. These analyses revealed that, of the variables in the cognitive reserve set, only mental activity was an independent predictor of reversion. For the sensory variables, both better smelling ability and better visual acuity independently predicted reversion. Of the set of health and genetic variables investigated, reversion was independently predicted by an absence of arthritis. The only independent predictor from among the personality variables was a greater level of openness. A number of independent predictors were identified from among the set of diagnostic variables. These included absences of either an informant-based memory complaint or multiple-domain MCI, and there not being moderate or severe impairment of a cognitive domain. The multivariable regression model for the neuroimaging set identified no significant effects, suggesting (on the basis of the univariate results) that left hippocampus and left amygdala volumes predicted reversion in a mutually dependent manner. Figure 2 summarises these findings and shows a Nagelkerke R^2^ value for each of the six multivariable regressions. These values approximate the relative importance of each set of variables for predicting reversion, and suggest that the set of diagnostic variables accounted for the largest amount of variability.

### Associations between Reversion and Longitudinal Change

Table 4 shows baseline and follow-up values for continuously-measured factors. Across all participants, there were significant decreases from baseline to follow-up in systolic and diastolic BP, cholesterol, and mental and physical activity, and a significant increase in homocysteine. A greater fall in diastolic BP for reverters than non-reverters was the only significant difference between these groups. As seen for categorically-measured factors in Table 5, social activity change patterns were similar but alcohol consumption increased in proportionally more reverters and decreased in proportionally more non-reverters. Regressions controlling for age and sex confirmed greater falls in diastolic BP and increased drinking (vs. unchanged drinking) as associated with significantly greater chances of reversion (odds ratio 1.03, 95% confidence interval 1.00–1.06, *p* = 0.026 and 3.25, 1.07–9.89, *p* = 0.038, respectively).

**Table 4:** Potentially modifiable continuously-measured characteristics of reverters and non-reverters at baseline and follow-up^a^.

Factor	Reverters (n = 64)^b^		Non-reverters (n = 156)^b^		*p* value	
	Baseline	Follow-up	Baseline	Follow-up	Time effect	Interaction
Systolic BP, mmHg	146.13 (18.86)	140.41 (18.78)	144.22 (20.15)	141.32 (19.74)	.006	.367
Diastolic BP, mmHg	82.94 (10.85)	77.82 (9.58)	81.40 (9.37)	80.05 (10.79)	<.001	.025
BMI, kg/m^2^	26.51 (4.00)	26.69 (4.26)	27.28 (4.60)	27.38 (4.64)	.307	.774
GDS score	2.37 (1.92)	2.34 (2.36)	2.20 (1.84)	2.51 (2.36)	.386	.280
Mental activity^c^	2.71 (0.82)	2.57 (0.93)	2.26 (0.84)	2.19 (0.77)	.043	.478
Physical activity^d^	1.54 (0.96)	1.36 (1.02)	1.65 (1.11)	1.38 (1.05)	<.001	.680
Cholesterol, mmol/L	4.66 (0.97)	4.62 (1.05)	4.85 (1.11)	4.57 (1.11)	.014	.060
Homocysteine, umol/L	11.43 (4.81)	13.40 (4.21)	12.17 (4.37)	13.52 (4.56)	<.001	.245

BP = blood pressure; BMI = body mass index; GDS = Geriatric Depression Scale.

a Data presented as mean (SD).

b Maximum n, with small amounts of data missing for some factors.

c Average days/week of participation in mental activities.

d No. physical activities participated in.

**Table 5:** Change in potentially modifiable categorically-measured characteristics and antihypertensive use from baseline to follow-up^a^.

Factor	All	Reverters	Non-reverters	*p* value^c^
	(n = 223)^b^	(n = 66)^b^	(n = 157)^b^	
***Alcohol consumption*** d				.014
Unchanged	182 (82.7)	54 (83.1)	128 (82.6)	
Increase	14 (6.4)	8 (12.3)	6 (3.9)	
Decrease	24 (10.9)	3 (4.6)	21 (13.5)	
***Social activity*** e				.590
Unchanged	126 (61.2)	40 (64.5)	86 (59.7)	
Increase	35 (17.0)	8 (12.9)	27 (18.8)	
Decrease	45 (21.8)	14 (22.6)	31 (21.5)	
***Antihypertensive use***				.047
Not used	73 (32.7)	27 (40.9)	46 (29.3)	
Baseline and follow-up	125 (56.1)	37 (56.1)	88 (56.1)	
Baseline only	9 (4.0)	0 (0.0)	9 (5.7)	
Follow-up only	16 (7.2)	2 (3.0)	14 (8.9)	

a Data presented as No. (%).

b Maximum n, with small amounts of data missing for some factors for either baseline or follow-up.

c Results comparing reverters and non-reverters.

d Change between abstainer, ≤1 drink/day, and >1 drink/day.

e Change between <5, 5–10, and >10 contacts/month.

The diastolic BP difference between reverters and non-reverters was further explored by analysing patterns of antihypertensive use. A significantly greater proportion of non-reverters than reverters had either stopped or begun using antihypertensives at follow-up; reverters were much more consistent in their use across baseline and follow-up (see Table 5).

## Discussion

We identified factors predicting or associated with reversion from MCI to normal cognition. Factors most indicative of prognosis were diagnostic features, with any of a diagnosis of multiple-domain MCI, moderate or severe impairment of a cognitive domain, or an informant-based memory complaint signalling a reduced chance of reversion. Arthritis was also associated with less reversion. Participants were more likely to revert if they had a greater level of mental activity, better control of BP, greater openness to experience, a larger left hippocampus/amygdala, better visual acuity, or better smelling ability.

### Interpretation of the Factors Associated with Reversion

The diagnosis of MCI requires an expressed concern (complaint) about memory and/or other cognitive difficulties, such as forgetfulness, inability to remember names, word-finding difficulties, getting lost, or difficulty in solving complex problems. The concern can be either self-reported or made by a knowledgeable informant. We found that reversion was less likely when concerns about a participant’s memory were expressed by an informant. This could be because concerns raised by friends or family members reflect more severe problems than self-reported concerns, which may arise from relatively minor age-related changes in cognitive functioning that are salient to the individual but unlikely to be perceived as pathological by others [36]. Supporting this idea is a previous report that self-reported complaints do not predict cognitive decline [37].

Our finding that individuals with multiple-domain MCI are less likely than those with single-domain MCI to revert to normal cognition is consistent both with previous reports [3], [13], [15]–[18] and with findings that multiple-domain MCI is more likely to transition to AD, which we have previously reported [38]. We also found that individuals with moderate or severe impairment in any one domain were less likely to revert. Other studies have similarly shown poorer cognitive performance to be associated with MCI that is either persistent or progressive [3], [14]–[16]. It is arguable that low cognitive performance not exceeding mild impairment may reflect temporary effects of extraneous factors such as depression, fatigue, poor motivation or intercurrent illness which are likely to change with time, prompting reconsideration of a cross-sectional diagnosis of MCI.

Our analysis of neuroimaging data revealed that reverters had significantly larger volumes of the left hippocampus and left amygdala than non-reverters. There have been previous reports of a non-significantly greater hippocampus volume in reverters than in non-reverters [39] and smaller volumes of the hippocampus, amygdala and caudate in MCI patients who progressed to AD than in patients with stable MCI or healthy controls [40]. Further, a recent meta-analysis found that MCI patients consistently showed less grey matter in the hippocampus and amygdala than healthy controls [41]. Our results support these structural changes as an element of stable or progressive MCI. It is unclear why we found effects for the left hemisphere only. Differences in right hippocampus and right amygdala volumes between reverters and non-reverters were apparent with simple analyses (see Table S2), but not when sex, age and intracranial volume were controlled for.

MCI reverters in our sample were less likely than non-reverters to have arthritis. This finding warrants cautious interpretation, given that the relationship between arthritis and AD is inconsistent [42]–[45] and complicated by the effect of pain on cognitive performance and the potential impact of long-term use of anti-inflammatory medication.

We also found that reverters reported more frequent engagement in mental activities like reading books, suggesting that they had a higher degree of brain or cognitive reserve than non-reverters. According to this conceptualization, individuals with high brain reserve have a greater buffer in the process of their decline before they reach a threshold for diagnosis of dementia [46]. The superior performance may be related to an efficient set of neural networks or a wider repertoire of conscious and preconscious cognitive strategies, and education enriches these. This repertoire arguably permits high reserve individuals to compensate for loss more effectively than those with a limited repertoire. There is also evidence that complex mental activity is protective against cognitive decline and the development of incident dementia [47]. The finding of higher brain reserve in the reverter group however is against expectation in one sense, as individuals with high reserve reportedly develop cognitive symptoms at a later stage of pathology than those with low reserve [48], and they should therefore be more likely to decline. We do not think that this was a factor in our study as normative data were corrected for education (where possible). Further, some individuals may have increased their level of cognitive activity after a perception of mild cognitive problems, and thereby reversed their deficits. The exact mechanism cannot be determined from the current data.

A greater level of cognitive reserve in reverters could help to account for some of our other findings, including the association between openness to experience and reversion. Openness may facilitate cognitive reserve by promoting active engagement in cognitively enriching activities that protect against cognitive decline [49]. The association between visual acuity and reversion can also be interpreted along these lines, with poor vision likely to prevent engagement in many cognitively enriching activities and thus limit the capacity to develop or maintain cognitive reserve. An alternative interpretation is that sensory loss is a marker for accelerated cognitive ageing with a greater likelihood of later developing AD [50]. Consistent with this idea is our finding that reverters also had better performance on a smell identification test. It has been previously found that individuals with MCI have poorer olfactory discrimination than controls [51]–[53], and that poorer performance on the BSIT is associated with an increased chance of progressing to AD [53]. Also previously demonstrated is a relationship between olfactory discrimination and AD pathology in the brain, even in individuals cognitively normal at the time of death [53]. Our olfactory identification and visual acuity findings support sensory loss as a marker for cognitive ageing.

The data also suggest that good control of BP is associated with increased likelihood of reversion. The reverters were more consistent in their antihypertensive drug usage and achieved a greater reduction in their diastolic BP over the two years. This is consistent with a previous finding [14] and reports that hypertension is associated with lower cognitive performance [54]. The relationship of diastolic BP to cognitive decline may be U-shaped, with both <60 mm Hg and >110 mm Hg associated with greater decline [55]. While antihypertensive treatment has been shown to decrease the risk of stroke, cardiovascular events and heart failure, the effect of the control of systolic BP on cognitive decline and the onset of dementia has been inconsistent [56]. More work is needed to examine the effect of the lowering of diastolic BP on cognitive decline in non-demented individuals.

The finding of an increase in alcohol consumption being associated with reversion was surprising, although tempered by the fact that in the majority (82.7%) of the sample, alcohol use was stable between baseline and follow-up. More reverters (12.3% vs. 3.9%) increased their alcohol use in this period (all from ≤1 to >1 drink/day), while more non-reverters (13.5% vs. 4.6%) decreased it (71.4% from >1 to ≤1 drink/day and 28.6% from ≤1 drink/day to abstainer). While there is evidence of the protective effect of moderate alcohol use against incident dementia, the published literatures is less clear on the effect on cognitive decline and predementia syndromes [57]. It is perhaps reasonable to conclude that light to moderate alcohol use is not deleterious in those with MCI, but the evidence is not persuasive enough to recommend initiation of alcohol use or increase in its quantity for the purpose of preventing decline or reversing MCI.

### Strengths and Limitations

Our study has numerous strengths, including the use of comprehensive assessment protocols at both baseline and follow-up, a large sample of participants with MCI at baseline, and a diverse range of factors investigated as potentially associated with reversion. No previous study appears to have simultaneously achieved all three of these. Our study also has limitations. The sample was population-based and participants diagnosed with MCI were not seeking help for cognitive difficulties. Individuals diagnosed with MCI in the clinic are likely to be a select group with potentially lower rates and different predictors of reversion. A number of individuals who had MCI at baseline were excluded from our analyses for missing a follow-up diagnostic classification. These individuals differed significantly from those remaining in the study in ways that suggest a reduced likelihood of reversion from MCI to normal cognition, including lower MMSE scores and mental activity and higher levels of neuroticism. Accordingly, we may be reporting an overestimated prevalence of reversion. An association between age and reversion was not found by us but has been reported for a sample with a mean age younger than ours [10]. Individuals younger than 70 years may show different predictors of reversion [14]. Further, our follow-up duration of two years provides only a narrow window into what can be a slow progression of neurodegenerative disease in older individuals. A longer follow-up is needed to determine the extent to which unstable MCI represents a very early MCI stage of serious cognitive decline. A final limitation is our lack of consideration for transitory cognitive impairment associated with factors like stress, acute illness or poor motivation, with MCI diagnosed under such conditions at greater than normal chances of reversion to normal cognition [10], [11], [15].

### Conclusions and Implications

A sizeable proportion of individuals categorised as MCI revert back to normal cognition. It is possible for reverters to have been misclassified initially, to have unstable MCI, or to have made true improvements in cognitive functioning upon follow-up. Knowing which individuals classified as MCI are more likely to revert to normal could help optimise the allocation of resources among MCI patients, with those considered least likely to revert receiving greater levels of intervention and follow-up contact. We have identified a number of diagnostic and other factors, albeit in a population-based sample, that may help determine if an MCI patient is likely to revert. Future research should aim to expand upon these findings by developing a predictive model that includes factors considered by clinicians as appropriate for routine use. For example, while MRI scans checking for hippocampus/amygdala atrophy are likely to be impractical, tests of visual acuity, olfactory identification, and personality might be suitably included in screening protocols. Our findings also suggest that continuing use of mild to moderate amounts of alcohol is not deleterious, and lead us to endorse both good control of BP and asking patients about their engagement in cognitively enriching activities. For patients with low engagement, activities suited to their interests, abilities and capacities should be identified and encouraged.

## Supporting Information

Table S1 **Cognitive domains, tests, normative data sources and demographic adjustments used in diagnosing MCI in the Sydney MAS.**
(DOCX)

Table S2 **Baseline region of interest volumes (mm^3^) for reverters and non-reverters in the MRI subsample.**
(DOCX)

Table S3 **Baseline characteristics of participants either with or without a diagnostic classification at follow-up.**
(DOCX)
