# Supplementary material for: Factors Predicting Reversion from Mild Cognitive Impairment to Normal Cognitive Functioning: A Population-Based Study
Source: PLoS One. 2013 Mar 27;8(3):e59649. doi: 10.1371/journal.pone.0059649 (PMC3609866; doi:10.1371/journal.pone.0059649)
Supplement: Table S1 — Cognitive domains, tests, normative data sources and demographic adjustments used in diagnosing MCI in the Sydney MAS. (DOCX) [file pone.0059649.s001.docx]

# Table S1. Cognitive domains, tests, normative data sources and demographic adjustments used in diagnosing MCI in the Sydney MAS

| **Cognitive Domain** | **Test** | **Normative Data Source & Demographic Adjustment(s)** |
| --- | --- | --- |
| **Premorbid Intelligence** | National Adult Reading Test (NART) [1] | No adjustment [1] |
| **Attention/Processing speed** | Digit-Symbol Coding [2] | Age [2] |
|  | Trail Making Test (TMT) A [3] | Age & education [4] |
| **Memory** | Logical Memory Story A delayed recall [5] | Education [6] |
|  | Rey Auditory Verbal Learning Test (RAVLT) [3] | Age [7] |
|  | RAVLT total learning (trials 1–5) |  |
|  | RAVLT short-term delayed recall (trial 6) |  |
|  | RAVLT long-term delayed recall (trial 7) |  |
|  | Benton Visual Retention Test recognition [8] | Age & education [9] |
| **Language** | Boston Naming Test (30 items) [10] | Age [11] |
|  | Semantic Fluency (Animals) [3] | Age & education [12] |
| **Visuospatial** | Block Design [13] | Age [14] |
| **Executive Function** | Controlled Oral Word Association Test (FAS) [3] | Age & education [12] |
|  | Trail Making Test (TMT) B [3] | Age & education [4] |

1. Nelson HE, Willison J. *National Adult Reading Test (NART): Test manual (2nd ed)*. Windsor, UK: NFER Nelson, 1991.

2. Wechsler D. *Wechsler Adult Intelligence Scale-III.* San Antonio, TX: The Psychological Corporation, 1997.

3. Strauss E, Sherman EMS, Spreen O. *A Compendium of Neuropsychological Tests: Administration, Norms, and Commentary (3rd ed)*. New York, NY: Oxford University Press, 2006.

4.Tombaugh TN. Trail Making Test A and B: Normative data stratified by age and education. *Arch Clin Neuropsychol* 2004;19:203-214.

5. Wechsler D. *Wechsler Memory Scale - 3rd Edition Manual*. San Antonio, TX: The Psychological Corporation, 1997.

6. Grundman M, Petersen RC, Ferris SH, Thomas RG, Aisen PS, Bennett DA, et al. Mild cognitive impairment can be distinguished from Alzheimer disease and normal aging for clinical trials. *Arch Neurol* 2004;61:59-66.

7. Ivnik RJ, Malec JF, Smith GE, Tangalos EG, Petersen RC, Kokmen E, et al. Mayo's Older Americans Normative Studies: updated AVLT norms for ages 56 to 97. *Clin Neuropsychol* 1992;6(suppl):83-104.

8. Benton AL, Sivan AB, Spreen O. *Der Benton Test [The Benton Test] (7th ed)*. Berne, Switzerland: Huber, 1996.

# 9. Lechevallier-Michel N, Fabrigoule C, Lafont S, Letenneur L, Dartigues JF. Normes por le MMSE, le Test de Retention Visuelle de Benton, le Set Test d’Isaacs (15s. et 60s), le sous-test des Codes de la WAIS et le Test Barrage de Zazzo, en function de l’age, du sexe et du niveau d’etudes, chez des sujets ages de 70 ans et plus: donnees de la chorte PAQUID [Normative data for the MMSE, the Benton visual retention test, the Isaacs's set test, the digit symbol substitution test and the Zazzo's cancellation task in subjects over the age 70: results from the PAQUID Study]. *Rev Neurol (Paris)* 2004;160:1059-1070.

# 10. Kaplan E. *The Boston Naming Test*. Philadelphia, PA: Lippincott Williams & Wilkins, 2001.

# 11. Fastenau PS, Denburg NL, Mauer BA. Parallel short forms for the Boston Naming Test: psychometric properties and norms for older adults. *J Clin Exp Neuropsychol* 1998;20:828-834.

12. Tombaugh TN, Kozak J, Rees L. Normative data stratified by age and education for two measures of verbal fluency: FAS and animal naming. *Arch Clin Neuropsychol* 1999;14:167-177.

13. Wechsler D. *WAIS-R Manual*. New York, NY: The Psychological Corporation, 1981.

14. Ivnik RJ, Malec JF, Smith GE, Tangalos EG, Petersen RC, Kokmen E, et al. Mayo's older Americans normative studies: WAIS-R norms for ages 56 to 97. *Clin Neuropsychol* 1992;6(suppl):1-30.
