# Supplementary material for: Factors Predicting Reversion from Mild Cognitive Impairment to Normal Cognitive Functioning: A Population-Based Study
Source: PLoS One. 2013 Mar 27;8(3):e59649. doi: 10.1371/journal.pone.0059649 (PMC3609866; doi:10.1371/journal.pone.0059649)
Supplement: Table S2 — Baseline region of interest volumes (mm3) for reverters and non-reverters in the MRI subsample. (DOCX) [file pone.0059649.s002.docx]

**Table S2. Baseline region of interest volumes (mm^3^) for reverters and non-reverters in the MRI subsample**

| **Region of interest** | **Reverters**  **(n = 37)** | | **Non-reverters**  **(n = 92)** | | ***p* value** |
| --- | --- | --- | --- | --- | --- |
|  | **Mean** | **SD** | **Mean** | **SD** |  |
| **Central region** |  |  |  |  |  |
| Precentral gyrus L | 7697 | 1205 | 7316 | 1353 | .138 |
| Precentral gyrus R | 7294 | 1185 | 7018 | 1359 | .282 |
| Postcentral gyrus L | 8151 | 1386 | 7783 | 1762 | .259 |
| Postcentral gyrus R | 7932 | 1262 | 7589 | 1583 | .241 |
| Rolandic operculum L | 2838 | 299 | 2725 | 490 | .113^a^ |
| Rolandic operculum R | 3694 | 387 | 367 | 663 | .816^a^ |
| **Frontal lobe** |  |  |  |  |  |
| *Lateral surface* |  |  |  |  |  |
| Superior frontal gyrus, dorsolateral L | 6971 | 1201 | 6706 | 1388 | .311 |
| Superior frontal gyrus, dorsolateral R | 8126 | 1266 | 7764 | 1497 | .198 |
| Middle frontal gyrus L | 11078 | 1993 | 10594 | 2043 | .223 |
| Middle frontal gyrus R | 11820 | 1804 | 11342 | 1969 | .204 |
| Inferior frontal gyrus, opercular part L | 2587 | 366 | 2485 | 437 | .210 |
| Inferior frontal gyrus, opercular part R | 3183 | 450 | 3152 | 529 | .753 |
| Inferior frontal gyrus, triangular part L | 5642 | 761 | 5514 | 1021 | .491 |
| Inferior frontal gyrus, triangular part R | 4794 | 696 | 4713 | 828 | .598 |
| *Medial surface* |  |  |  |  |  |
| Superior frontal gyrus, medial L | 6677 | 1019 | 6409 | 1096 | .202 |
| Superior frontal gyrus, medial R | 5077 | 863 | 4868 | 906 | .232 |
| Supplementary motor area L | 4843 | 665 | 4594 | 823 | .104 |
| Supplementary motor area R | 5187 | 787 | 4984 | 920 | .240 |
| Paracentral lobule L | 2653 | 473 | 2524 | 562 | .223 |
| Paracentral lobule R | 1828 | 355 | 1704 | 368 | .083 |
| *Orbital surface* |  |  |  |  |  |
| Superior frontal gyrus, orbital part L | 2487 | 374 | 2381 | 394 | .162 |
| Superior frontal gyrus, orbital part R | 2481 | 426 | 2370 | 386 | .152 |
| Superior frontal gyrus, medial orbital L | 1890 | 346 | 1813 | 328 | .197 |
| Superior frontal gyrus, medial orbital R | 2479 | 430 | 2333 | 410 | .122 |
| Middle frontal gyrus , orbital part L | 2468 | 421 | 2358 | 441 | .239 |
| Middle frontal gyrus , orbital part R | 2848 | 537 | 2694 | 498 | .075 |
| Inferior frontal gyrus, orbital part L | 4962 | 728 | 4835 | 795 | .405 |
| Inferior frontal gyrus, orbital part R | 4771 | 716 | 4700 | 726 | .610^a^ |
| Gyrus rectus L | 2573 | 372 | 2471 | 432 | .209 |
| Gyrus rectus R | 2372 | 413 | 2212 | 367 | .034 |
| Olfactory cortex L | 1036 | 151 | 985 | 161 | .098 |
| Olfactory cortex R | 1055 | 146 | 1013 | 168 | .187 |
| **Temporal lobe** |  |  |  |  |  |
| *Lateral surface* |  |  |  |  |  |
| Superior temporal gyrus L | 7882 | 1037 | 7441 | 1279 | .064 |
| Superior temporal gyrus R | 8316 | 999 | 8043 | 1394 | .217^a^ |
| Heschl gyrus L | 779 | 137 | 717 | 144 | .028 |
| Heschl gyrus R | 644 | 86 | 602 | 127 | .030^a^ |
| Middle temporal gyrus L | 14458 | 1767 | 13968 | 2071 | .208 |
| Middle temporal gyrus R | 13376 | 1609 | 12828 | 2116 | .158 |
| Inferior temporal gyrus L | 9655 | 1341 | 9250 | 1547 | .166 |
| Inferior temporal gyrus R | 10501 | 1487 | 10041 | 1662 | .146 |
| **Parietal lobe** |  |  |  |  |  |
| *Lateral surface* |  |  |  |  |  |
| Superior parietal gyrus L | 5316 | 1054 | 4991 | 1035 | .112 |
| Superior parietal gyrus R | 5106 | 860 | 4790 | 967 | .086 |
| Inferior parietal (but supramarginal and angular gyri L) | 6439 | 968 | 6289 | 1245 | .511 |
| Inferior parietal (but supramarginal and angular gyri R) | 3610 | 489 | 3510 | 716 | .439 |
| Angular gyrus L | 3580 | 541 | 3487 | 642 | .438 |
| Angular gyrus R | 4866 | 622 | 4746 | 909 | .391^a^ |
| Supramarginal gyrus L | 3236 | 522 | 3260 | 676 | .848 |
| Supramarginal gyrus R | 4712 | 648 | 4703 | 925 | .950^a^ |
| *Medial surface* |  |  |  |  |  |
| Precuneus L | 9455 | 1460 | 8980 | 1492 | .102 |
| Precuneus R | 8591 | 1210 | 8169 | 1344 | .100 |
| **Occipital lobe** |  |  |  |  |  |
| *Lateral surface* |  |  |  |  |  |
| Superior occipital gyrus L | 3285 | 587 | 3083 | 570 | .073 |
| Superior occipital gyrus R | 3340 | 620 | 3218 | 545 | .268 |
| Middle occipital gyrus L | 9429 | 1238 | 8968 | 1492 | .098 |
| Middle occipital gyrus R | 5833 | 854 | 9429 | 1238 | .524 |
| Inferior occipital gyrus L | 2862 | 410 | 2739 | 427 | .139 |
| Inferior occipital gyrus R | 2767 | 455 | 2732 | 535 | .726 |
| *Medial and inferior surfaces* |  |  |  |  |  |
| Cuneus L | 4253 | 768 | 4087 | 737 | .256 |
| Cuneus R | 4343 | 859 | 4196 | 690 | .311 |
| Calcarine fissure and surrounding cortex L | 6757 | 1135 | 6383 | 1170 | .101 |
| Calcarine fissure and surrounding cortex R | 5459 | 898 | 5234 | 959 | .222 |
| Lingual gyrus L | 6906 | 922 | 6555 | 1001 | .068 |
| Lingual gyrus R | 6507 | 912 | 6266 | 992 | .206 |
| Fusiform gyrus L | 8618 | 1140 | 8079 | 1219 | .022 |
| Fusiform gyrus R | 8197 | 1158 | 8618 | 1140 | .258 |
| **Limbic lobe** |  |  |  |  |  |
| Temporal pole: superior temporal gyrus L | 3748 | 618 | 3501 | 530 | .024 |
| Temporal pole: superior temporal gyrus R | 3807 | 538 | 3648 | 577 | .150 |
| Temporal pole: middle temporal gyrus L | 2185 | 404 | 2077 | 411 | .178 |
| Temporal pole: middle temporal gyrus R | 3102 | 500 | 3051 | 615 | .651 |
| Anterior cingulate and paracingulate gyri L | 4120 | 659 | 4036 | 657 | .516 |
| Anterior cingulate and paracingulate gyri R | 3950 | 548 | 3901 | 633 | .681 |
| Median cingulate and paracingulate gyri L | 5284 | 578 | 5068 | 738 | .113 |
| Median cingulate and paracingulate gyri R | 5877 | 587 | 5690 | 831 | .153^a^ |
| Posterior cingulate gyrus L | 1147 | 216 | 1063 | 201 | .038 |
| Posterior cingulate gyrus R | 728 | 121 | 683 | 121 | .055 |
| Hippocampus L | 3519 | 365 | 3281 | 451 | .005 |
| Hippocampus R | 3208 | 401 | 3045 | 428 | .048 |
| Parahippocampal gyrus L | 3652 | 486 | 3407 | 519 | .015 |
| Parahippocampal gyrus R | 3840 | 504 | 3640 | 553 | .059 |
| Insula L | 5916 | 720 | 5494 | 828 | .222 |
| Insula R | 5610 | 760 | 4036 | 657 | .463 |
| **Subcortical grey nuclei** |  |  |  |  |  |
| Amygdala L | 868 | 112 | 787 | 125 | .001 |
| Amygdala R | 908 | 124 | 846 | 133 | .016 |
| Caudate nucleus L | 3278 | 455 | 3006 | 572 | .011 |
| Caudate nucleus R | 3399 | 462 | 3153 | 550 | .018 |
| Lenticular nucleus, putamen L | 2662 | 579 | 2423 | 505 | .021 |
| Lenticular nucleus, putamen R | 2701 | 596 | 2505 | 522 | .065 |
| Lenticular nucleus, pallidum L | 261 | 74 | 267 | 95 | .753 |
| Lenticular nucleus, pallidum R | 214 | 70 | 222 | 82 | .631 |
| Thalamus L | 2485 | 368 | 2361 | 383 | .098 |
| Thalamus R | 2165 | 395 | 2078 | 370 | .240 |
| **Cerebellum lobule** |  |  |  |  |  |
| Crus I L | 8850 | 1339 | 8376 | 1404 | .082 |
| Crus I R | 7893 | 1222 | 7445 | 1302 | .075 |
| Crus II L | 7148 | 974 | 6739 | 1106 | .052 |
| Crus II R | 7252 | 1102 | 6808 | 1084 | .039 |
| III L | 433 | 62 | 418 | 67 | .218 |
| III R | 569 | 70 | 551 | 83 | .247 |
| IV,V L | 3758 | 459 | 3685 | 578 | .497 |
| IV,V R | 2824 | 361 | 2735 | 425 | .266 |
| VI L | 5613 | 780 | 5386 | 950 | .200 |
| VI R | 5550 | 749 | 5392 | 883 | .342 |
| VIIB L | 1959 | 257 | 1851 | 315 | .067 |
| VIIB R | 1814 | 279 | 1683 | 282 | .017 |
| VIII L | 6249 | 804 | 5923 | 939 | .066 |
| VIII R | 6528 | 936 | 6099 | 948 | .021 |
| IX L | 2938 | 506 | 2780 | 437 | .079 |
| IX R | 2594 | 445 | 2424 | 409 | .039 |
| X L | 324 | 57 | 302 | 52 | .037 |
| X R | 295 | 62 | 277 | 47 | .069 |
| **Vermis lobule** |  |  |  |  |  |
| I,II | 115 | 16 | 110 | 18 | .167 |
| III | 584 | 73 | 564 | 89 | .233 |
| IV,V | 1765 | 195 | 1733 | 262 | .505 |
| VI | 1234 | 143 | 1193 | 166 | .194 |
| VII | 708 | 97 | 677 | 107 | .133 |
| VIII | 810 | 111 | 769 | 135 | .101 |
| IX | 581 | 92 | 560 | 95 | .254 |
| X | 144 | 20 | 140 | 23 | .351 |

SD = standard deviation; L = left hemisphere; R = right hemisphere.

^a^ *t*-test for unequal variances.
