# Supplementary material for: Factors Predicting Reversion from Mild Cognitive Impairment to Normal Cognitive Functioning: A Population-Based Study
Source: PLoS One. 2013 Mar 27;8(3):e59649. doi: 10.1371/journal.pone.0059649 (PMC3609866; doi:10.1371/journal.pone.0059649)
Supplement: Table S3 — Baseline characteristics of participants either with or without a diagnostic classification at follow-up. (DOCX) [file pone.0059649.s003.docx]

**Table S3. Baseline characteristics of participants either with or without a diagnostic classification at follow-up**^a^

| **Factor** | **Classified**  **(n = 234)^b^** | **No classification**  **(n = 86)^b^** | ***p* value** |
| --- | --- | --- | --- |
| **Sociodemographic** |  |  |  |
| Age, mean (SD), y | 78.62 (4.43) | 80.08 (5.22) | .01 |
| Males | 116 (49.6) | 37 (43.0) | .29 |
| Education, mean (SD), y | 11.63 (3.72) | 11.33 (3.38) | .51 |
| Married or de facto | 99 (42.5) | 27 (31.4) | .07 |
| **Cardiac Health** |  |  |  |
| Hypertension | 192 (82.1) | 71 (82.6) | .91 |
| Antihypertensives | 139 (59.4) | 57 (66.3) | .26 |
| Coronary artery disease | 44 (18.8) | 21 (24.4) | .26 |
| Atrial fibrillation | 17 (7.4) | 9 (10.8) | .33 |
| Other heart disease^c^ | 29 (12.4) | 17 (19.8) | .09 |
| Systolic BP, mean (SD), mmHg | 144.79 (19.8) | 139.50 (21.76) | .04 |
| Diastolic BP, mean (SD), mmHg | 81.78 (9.99) | 79.58 (11.26) | .09 |
| **Physical Health** |  |  |  |
| BMI, mean (SD), kg/m^2^ | 27.02 (4.40) | 26.10 (4.40) | .10 |
| Diabetes | 35 (15.0) | 8 (9.3) | .18 |
| Hypoglycemics | 22 (90.6) | 6 (7.0) | .49 |
| High cholesterol diagnosis | 131 (56.0) | 52 (60.5) | .47 |
| Hypolipidemics | 110 (47.0) | 44 (51.2) | .51 |
| Stroke | 11 (4.7) | 5 (6.0) | .65 |
| Migraines | 30 (12.8) | 12 (14.0) | .79 |
| Kidney disease | 8 (3.4) | 2 (2.4) | .62 |
| Arthritis | 133 (57.3) | 43 (52.4) | .44 |
| Apnea | 10 (4.3) | 7 (8.1) | .17 |
| Anemia | 26 (11.2) | 8 (9.4) | .64 |
| **Mental Health** |  |  |  |
| GDS score, mean (SD) | 2.21 (1.82) | 2.75 (2.36) | .06^d^ |
| History of depression | 36 (15.4) | 16 (18.6) | .48 |
| GAS score, mean (SD) | 1.22 (2.02) | 1.08 (1.87) | .58 |
| Antidepressants | 21 (9.0) | 10 (11.6) | .47 |
| Antianxiety agents | 11 (4.7) | 7 (8.1) | .23 |
| **Lifestyle** |  |  |  |
| Alcohol consumption |  |  | .02 |
| Abstainer | 23 (9.8) | 16 (18.6) |  |
| ≤ 1 drink/day | 118 (50.4) | 47 (54.7) |  |
| > 1 drink/day | 93 (39.7) | 23 (26.7) |  |
| Smoking |  |  | .75 |
| Never | 110 (47.0) | 44 (51.2) |  |
| Past | 113 (48.3) | 39 (45.3) |  |
| Current | 11 (4.7) | 3 (3.5) |  |
| Mental activity, mean (SD)^e^ | 2.38 (0.84) | 2.17 (0.79) | .04 |
| Physical activity, mean (SD)^f^ | 1.60 (1.10) | 1.29 (0.96) | .02 |
| Social activity |  |  | .18 |
| < 5 (contacts/month) | 29 (12.7) | 17 (20.5) |  |
| 5 to 10 (contacts/month) | 57 (25.0) | 22 (26.5) |  |
| > 10 (contacts/month) | 142 (62.3) | 44 (53.0) |  |
| **General Health** |  |  |  |
| Self-reported |  |  | .03 |
| Poor to fair | 32 (13.7) | 19 (22.1) |  |
| Good | 93 (39.7) | 40 (46.5) |  |
| Very good to excellent | 109 (46.6) | 27 (31.4) |  |
| 6-m walk time, mean (SD), s | 9.50 (2.83) | 10.05 (3.20) | .14 |
| BSIT score, mean (SD) | 8.98 (2.28) | 8.78 (2.24) | .49 |
| Visual acuity, mean (SD)^g^ | 0.66 (0.20) | 0.69 (0.23) | .30 |
| **Laboratory Measures** |  |  |  |
| Apolipoprotein E ε4 allele | 66 (28.8) | 15 (21.1) | .20 |
| Homocysteine, mean (SD), umol/L | 12.31 (4.97) | 11.81 (3.79) | .43 |
| Cholesterol, mean (SD), mmol/L | 4.75 (1.06) | 4.74 (0.98) | .93 |
| eGFR < 60 ml/min/1.73 m^2^ | 89 (39.7) | 32 (45.1) | .42 |
| **Diagnostic characteristics** |  |  |  |
| MMSE score^h^ | 28 .25 (1.44) | 27.81 (1.57) | .02 |
| Bayer ADL Scale score | 1.53 (0.57) | 1.61 (0.53) | .31 |
| Memory complaint |  |  | .52 |
| Informant, no. (%) | 158 (71.5) | 51 (66.2) |  |
| Self-report only, no. (%) | 58 (26.2) | 25 (32.5) |  |
| Non-memory complaint |  |  | .007 |
| Informant, no. (%) | 77 (34.8) | 35 (45.5) |  |
| Self-report only, no. (%) | 102 (46.2) | 20 (26.0) |  |
| Amnestic MCI |  |  | .24 |
| No, no. (%) | 107 (45.7) | 33 (38.4) |  |
| Yes, no. (%) | 127 (54.3) | 53 (61.6) |  |
| Multiple-domain MCI |  |  | .36 |
| No, no. (%) | 159 (67.9) | 54 (62.8) |  |
| Yes, no. (%) | 75 (32.1) | 32 (37.2) |  |
| Performance in worst domain |  |  | .53 |
| Low, no. (%) | 55 (24.9) | 15 (19.5) |  |
| Mildly impaired, no. (%) | 72 (32.6) | 23 (29.9) |  |
| Moderately impaired, no. (%) | 59 (26.7) | 22 (28.6) |  |
| Severely impaired, no. (%) | 35 (15.8) | 17 (22.1) |  |
| **Brain region volumes** | **(n = 133)** | **(n = 42)** |  |
| Grey matter, l | 0.74 (0.10) | 0.72 (0.09) | .42 |
| White matter, l | 0.38 (0.04) | 0.37 (0.04) | .17 |
| Total brain volume, l | 1.11 (0.13) | 1.09 (0.12) | .27 |
| Cerebrospinal fluid, l | 0.44 (0.12) | 0.41 (0.09) | .06^d^ |
| Intracranial volume, l | 1.56 (0.19) | 1.50 (0.18) | .09 |
| WMH, mm^3^ | 10838 (17562) | 16232 (22800) | .10 |
| Region of interest, mm^3^ |  |  |  |
| Hippocampus (left) | 4451 (557) | 4353 (614) | .33 |
| Amygdala (left) | 836 (114) | 819 (146) | .43 |
| Caudate (left) | 2916 (487) | 2793 (479) | .15 |
| Caudate (right) | 2873 (465) | 2755 (485) | .16 |
| Putamen (left) | 3491 (456) | 3489 (472) | .97 |
| Cerebellum 7b (right) | 1723 (292) | 1638 (403) | .14 |
| Cerebellum 8 (right) | 6229 (975) | 6073 (1421) | .50^d^ |
| **Personality scale scores** | **(n = 143)** | **(n = 40)** |  |
| Neuroticism | 14.41 (6.55) | 17.00 (8.22) | .03 |
| Openness | 26.01 (5.89) | 25.73 (6.70) | .79 |
| Conscientiousness | 34.03 (5.77) | 33.85 (7.80) | .88^d^ |

ADL = Activity of Daily Living; BMI = body mass index; BP = blood pressure; BSIT= Brief Smell Identification Test; eGFR = estimated glomerular filtration rate; GAS = Goldberg Anxiety Scale; GDS = Geriatric Depression Scale; MCI = mild cognitive impairment; MMSE = Mini-Mental State Examination; WMH = white matter hyperintensities.

^a^ Data presented as no. (%) unless otherwise indicated.

^b^ Maximum n, with small amounts of missing data for some factors.

^c^ Any of cardiac arrhythmia, cardiomyopathy, or heart valve disease.

^d^ Result for *t*-test for unequal variances.

^e^ Average days/week of participation in mental activities.

^f^ Number of different physical activities participated in.

^g^ Arbitrary units, averaged for the two eyes.

^h^ Adjusted for age and education.
